# A novel anti-DR5 antibody-drug conjugate possesses a high-potential therapeutic efficacy for leukemia and solid tumors

**DOI:** 10.7150/thno.33598

**Published:** 2019-07-13

**Authors:** Shuyong Zhang, Chao Zheng, Wan Zhu, Peng Xiong, Dongdong Zhou, Changjiang Huang, Dexian Zheng

**Affiliations:** 1 Yantai Obioadc Biomedical Technology Ltd., Yantai, China; 2 Obio Technology (Shanghai) Corp., Ltd., No. 908, Bldg. 19, Ziping Rd., Pudong New District, Shanghai 201321, China; 3 Yantai Mabplex International Bio-Pharmaceutical Co., Ltd., Yantai, China

**Keywords:** DR5 antibody, antibody-drug conjugate, leukemia, solid tumor, therapy

## Abstract

It is well known that tumor necrosis factor-related apoptosis inducing ligand receptor 1 or 2 (DR4/DR5) is specifically expressed in various tumor cells, but less or no expression in most normal cells. Many first generations of TRAIL agonists including recombinant preparations of TRAIL, agonistic antibodies against DR4/DR5 have been developed in phase I/II clinical trials for cancer therapy. However, the outcomes of clinical trials by using DR4/DR5 agonist mono-therapy were disappointed even though the safety profile was well tolerance. In the present study, we report an anti-DR5 antibody-drug conjugate (ADC, named as Zapadcine-1) possesses a higher potential for the therapy of lymphocyte leukemia and solid cancers.

**Methods:** Zapadcine-1 was made by a fully humanized DR5-specific monoclonal antibody (Zaptuzumab) coupled via a cleavable linker to a highly toxic inhibitor of tubulin, monomethyl auristatin D (MMAD), by using ThioBridge technology. Cytotoxicity of the ADC in various tumor cells was identified by luminescent cell viability assay and the efficacy *in vivo* was determined in cells derived xenografts (CDX) of Jurkat E6-1, BALL-1, Reh, and patient derived xenografts (PDX) of human acute leukemia. Preliminary safety evaluation was carried out in rat and monkey. **Results:** Zapadcine-1 possesses a similar binding ability to the death receptor DR5 as the naked monoclonal antibody Zaptuzumab, and can be rapidly endocytosed into the lysosome of cancer cells. Zapadcine-1 specifically kills human lymphocyte leukemia cells and solid tumor cells, but not normal cells tested. More importantly, Zapadcine-1 drastically eliminates the xenografts in both CDX and PDX models of human acute leukemia. The excellent and comparable therapeutic efficacy is also observed in lung cancer NCI-H1975 CDX mouse model. The maximum-tolerated dose (MTD) of single injected Zapadcine-1 in rat and cynomolgus monkey shows an acceptable safety profile.

**Conclusion:** These data demonstrate a promising anti-cancer activity, meriting further exploration of its potential as a novel cancer therapeutic agent, especially for the acute lymphocyte leukemia.

## Introduction

Tumor necrosis factor-related apoptosis-inducing ligand (TRAIL) induces cell death specifically in about 40-50% of tumor cells via an extrinsic or intrinsic signaling pathway, but not in most normal cells 1-4. Up to date, several first generation TRAIL receptor-targeting agonists including recombinant soluble TRAIL (rsTRAIL), agonistic monoclonal antibody against TRAIL-R1 (DR4) or TRAIL-R2 (DR5) have been developed for cancer therapy in early phase clinical trials (phase I/II) 5. However, the single drug monotherapy is far from prospective, even though the safety profile is well tolerance 6-9. It is believed that the poor performance of death receptor agonists in clinical trials are caused by the shorter *in vivo* half-life of rsTRAIL, the lower agonistic activity of the antibodies, more complexity of death receptors (DR4, DR5) mediated signaling pathways, therefore, no proper molecular markers available for the clinical patient selection 10-12. Current study and development of the antitumor drugs targeting DR4/DR5 are focusing on increasing both bioactivity and efficacy of these agonists 13-15. And progresses have been made in these aspects by combination therapy with chemotherapeutics, radiotherapy, antibodies, inhibitors, as well as carrying drug's nanoparticles. The preliminary combination therapy in clinical trials demonstrates an improving overall response and survival in certain solid tumors 16. Meanwhile, second generation of DR4/DR5 agonists, such as rsTRAIL fused to Fc, single chain of monoclonal antibodies functionalized to nanoparticles or linkers, in order to increase their polymerization, have developed with aims to increase both bioavailability and efficacy 8, 17. Preliminary results have been reported in preclinical or early clinical trials 18, 19, but intensive clinical trials are needed to be achieved.

In comparison, antibody drugs have much tumor specific killing activity and better safety profile. Especially, antibody-drug conjugates (ADC) bind to the tumor-related antigen on the cell surface could be endocytosed into lysosome and then the toxin payload coupled to the antibody released to kill tumor cells specifically. So that ADC can increase therapeutic efficacy, reduce side effect, thus becomes a rising star in development of cancer therapeutics 20-24.

The payloads in ADCs are mainly composed of the chemical toxins including tubulin polymerase inhibitors, such as monomethyl auristatin E (MMAE), F (MMAF) and D (MMAD). Up to date, many ADCs uses MMAE and MMAF as payloads, but there is no ADCs coupled with MMAD in clinic or clinical trials up to date, which might be involved in the less stability of MMAD metabolism in mouse plasma than that in rat, monkey and human 25, therefore, effect on ADC efficacy and toxicity. Maytansine (DM1 and DM4) and those disrupt DNA double-helix structure, such as calicheamicin and carcinomycin, are also currently used in ADC composition for clinical trials 26.

It has been previously reported that a mouse mAb, AD5-10, specifically binds to the extracellular domain of human DR5 and induces apoptosis and autophagic cell death in various tumor cell lines and the xenografts of human liver, lung, colon, breast, and ovarian cancers 27-31. The humanized monoclonal antibody of AD5-10 (named as Zaptuzumab) possesses both apoptotic and autophagic cell death-inducing activity as the parental antibody. Furthermore, Zaptuzumab possesses two important features, i.e., cancer specificity and could be endocytosed into lysosome rapidly 13. These two features lay an excellent foundation for ADC drug development. In the present study, we synthesized a series of Zaptuzumab-linker-toxin conjugates using ThioBridge technology 32 and screened out an ADC (named as Zapadcine-1) coupled with a cleavable linker-MMAD possessing much higher potential as a novel therapeutics for lymphocyte leukemia and solid cancers.

## Materials and Methods

### Cell lines and animals

Acute T lymphocytic leukemia cells (Jurkat E6-1, J.gammal and A3), acute B lymphocytic leukemia cells (BALL-1), acute monocytic leukemia cells (THP-1), acute lymphocytic leukemia cells (Reh, non T and non B), non-small cell lung cancer cells (NCI-H1975 and MSTO-211H) were purchased from the Cell Bank of Chinese Academy of Medical Sciences (Beijing, China), Cell Bank of Chinese Academy of Shanghai Institutes for Biological Sciences (Shanghai, China) and ATCC (American Type Culture Collection, Manassas, VA), respectively, and cultured in RPMI-1640 medium or Dulbecco's modified Eagle's medium (Gibco) supplemented with 10% fetal bovine serum (Gibco) and 1% penicillin/streptomycin (North China Pharmaceutical Co., Shijiazhuang, China). All cell lines were cultured in a humidified incubator (Thermo Fisher Scientific, Waltham, MA), and maintained at 37 ℃ with 5% CO_2_.

NOD/SCID mice or BALB/c nude mice (5-6 weeks old) were purchased from Shanghai Laboratory Animal Center (Shanghai, China). All mice were housed and maintained in the specific pathogen free (SPF) grade of animal care facility. Five to six weeks old mice at the experimental initiation were maintained with standard laboratory chow and water ad libitum. All animal experiments were approved and performed in full compliance with guidelines approved by the Animal Care Committee of the Center for the Experimental Animals, Obio Technology (Shanghai) Corp., Ltd.

### Antibody and ADC synthesis

The humanized anti-DR5 antibody (Zaptuzumab) 13 was used for ADC preparation. Zaptuzumab protein was partially reduced with Tris (2-carboxyethyl) phosphine hydrochloride (TCEP). Then the buffer was exchanged by elution through Sephadex G-25 resin column with PBS containing 1.0 mM diethylenetriamine pentaacetic acid (DTPA). The conjugation reaction mixture was prepared by adding dimethyl sulfoxide (DMSO) to the reduced antibody solution. The linker-toxin was synthesized and dissolved in DMSO, and then mixed rapidly with one volume of conjugation buffer containing 4.4 moles of drug linkers and one mole of naked antibody, then placed on ice for 1.0 h followed by centrifugal ultrafiltration and buffer exchange with Sephadex G25 chromatography and PBS elution. The ADC conjugates were then filtered through a 0.2 μm filter under sterile conditions and stored at -80 ℃^33^. The ADCs were analyzed by HIC (hydrophobic interaction chromatography) (1200 HPLC, Agilent, Wilmington, DE, USA). The identification for peaks corresponding to ADCs with two, four, and six moles of toxin per mole antibody was accomplished using the method previously reported by Hamblett et al. 34. The drug/Ab ratio was determined by peak area integration.

### ELISA

High binding 96-well plates were coated with the recombinant DR5 (Sino Biological Inc., Beijing, China) and blocked with 2% BSA/PBS/0.05% Tween-20 solution. Zapadcine-1 and the naked antibody Zaptuzumab at varying concentrations were added respectively in the wells and incubated at 37 °C for 1.0 h. The wells were washed with PBS containing 0.05% Tween-20 (PBST) and incubated with HRP-labeled donkey anti-human IgG-H&L (Abcam, ab102438) at 37 °C. The excess probe was washed from the wells with PBST. TMB (3,3',5,5'-Tetramethylbenzidine) substrate solution (Solarbio, Shanghai, China, PR1200) was added and incubated at room temperature for an appropriate time (usually 5 to 10 min) followed by adding stop solution (1.0 M H_2_SO_4_). The optical density at 450 nm was determined on SPARK 10M multiplate reader (TECAN, 1703004862, Switzerland).

### Flow cytometry analysis

Cancer cells of Jurkat E6-1, BALL-1, Reh, NCI-H1975, MSTO-211H, and normal cells of PBMC, BEAS-2B and WRL68 (2×10^5^ cells/mL) were cultured in proper media to the logarithmic growth phase. Suspended grow cells were collected by centrifugation. Adherent grow cells were trypsinized first and then collected by centrifugation. Cells were re-suspended in culture media without FBS and incubated with 10 μg/mL Zapadcine-1 or anti-human IgG antibody (Abcam, ab200699) for 2 h on ice, then rinsed with ice-cold PBS. The cells were fixed with 4% paraformaldehyde for 10 min, and permeabilized with 0.2% Triton X-100 in PBS for 5 min at room temperature followed by incubation with 1.0 μg/mL of goat anti-human IgG Alexa Fluor 488-conjugated F(ab')_2_ fragment (Jackson ImmunoResearch) at 37 °C for 30 min, then washed with PBST. The labeled cells were washed and resuspended in PBS, and then the cell-associated fluorescence was determined by FACS Calibur (BD Biosciences, San Jose, CA, USA).

### Internalization assay

Approximately 5×10^4^ cells of MSTO-211H in the logarithmic growth phase were added on the Lab-Tek chambered cover glass (Thermo Fisher Scientific, Waltham, MA). Next day, the cells were stained with 20 μg/mL of fluoresceine isothiocyanate (FITC)-labeled Zapadcine-1 or 1.0 mL of lysosome specific dye of Lyso-Tracker Red (Thermo Fisher Scientific, Waltham, MA) at 1:10,000 dilution in PBS for 0.0 h, 0.5 h and 1.0 h at room temperature, then washed with PBS, covered with the coverslip. Images were captured by the laser scanning confocal microscopy (ZEISS LSM710, Germany).

### *In vitro* cytotoxicity assay

The cytotoxicity of the Zapadcine-1 was determined by CellTiter-Glo® Luminescent Cell Viability Assay (Promega, G7572) according to the manufacturer's instructions. Briefly, tumor cells and normal cells were seeded into 96-well plates at 2.5×10^4^ cells per well in 100 μL complete medium, then incubated at 37 °C with 5% CO_2_ overnight. Untreated cells served as control. Zapadcine-1 or Zaptuzumab in 100 μL medium were added in triplicate at various concentrations, respectively, and incubated for 48 h. Absorbance was measured at 450 nm by SPARK 10M Multiplate Reader (TECAN, 1703004862, Switzerland). The cell survival rate (%) was calculated using the following formula: A_sample_/A_control_×100%. The 50% inhibitory concentration (IC50) was calculated by SPSS software.

### Preliminary toxicity of Zapadcine-1 in animals

To investigate the preliminary toxicity *in vivo* and tolerability of Zapadcine-1, MTD study was conducted in SD rats and cynomolgus monkeys. Rats were single-dosed intravenously on day 1 with vehicle, 10 mg/kg, 20 mg/kg, 30 mg/kg, or 40 mg/kg of Zapadcine-1, respectively (n = 4). Cynomolgus monkeys were single-dosed intravenously on days 1 with vehicle, 2.0 mg/kg, 3.0 mg/kg, 4.0 mg/kg or 5.0 mg/kg of Zapadcine-1, respectively (n = 1) 35, 36. Assessment of toxicity was based on mortality, clinical signs, food consumption, body weight, clinical and anatomic pathology. Necropsy was performed and tissues were routinely processed.

### *In vivo* therapeutic efficacy

NOD/SCID mice and BALB/c nude mice (5-6 weeks old) were purchased from Shanghai Laboratory Animal Center (Shanghai, China). The mice were housed and maintained under SPF condition. All animal experiments were approved and performed in full compliance with guidelines approved by the Animal Care Committee of the Center for the Experimental Animals, Obio Technology (Shanghai) Corp., Ltd. Five to six weeks old mice at the experimental initiation were maintained on standard laboratory chow and water ad libitum. For the xenograft models, 1×10^7^ Jurkat E6-1 or BALL-1 or Reh cells, 5×10^6^ NCI-H1975 cells suspended in 200 μL PBS were injected subcutaneously into the right flank of mice. When the tumor volume reached to 100 to 200 mm^3^, the mice were randomized into groups and injected intravenously with Zapadcine-1 at 1.0 mg/kg, 3.0 mg/kg or 9.0 mg/kg every three days for a total of three injections (Q3D×3, every three days for 3 times). Physiological saline was administered as vehicle control. Tumor sizes were measured twice a week with a caliper, and tumor volumes were determined according to the formula: tumor volume (mm^3^) = longer diameter × (shorter diameter)^2^ × 0.5. The inhibition rate of tumor growth was calculated as [1 - (tumor volume treated final - tumor volume treated initial)/(tumor volume control final - tumor volume control initial)] × 100%. At the end of the experiment, mice were euthanized. For the T lymphocyte leukemia PDX model, HuKemia^®^ AL7442 cells (Crown Bioscience, Taichang, China) were thaw out from liquid nitrogen in a 37 °C water-bath rapidly, and transferred into a 50 mL Falcon tube with 40 mL pre-warmed complete medium. The cells were pelleted by centrifugation at 1000 rpm for 7 min, and washed twice with ice cold PBS, then re-suspended into ice cold PBS. The cells (8×10^5^) were intravenously injected into NOD/SCID mice for tumor development. After inoculation, eye blood was weekly collected from the animals and the human CD45^+^ cells in the mouse PBMC were stained and determined by FACS to monitor tumor burden. When average of tumor burden reached about 4%, the mice were randomly allocated into 4 groups (n = 3). The day of grouping and dosing was denoted as day 0. The mice were intravenously received 1.0 mg/kg, 3.0 mg/kg and 9 mg/kg of Zapadcine-1 (n = 3) at day 0, day 4 and day 7, respectively. Physiological saline was administered as the vehicle control. The tumor burden of each groups were determined and analyzed accordingly.

### Statistical analysis

Data of all experiments were presented as mean values ± standard deviations (SD) by Graphpad Prism 5 software. IC50 values were determined by nonlinear regression analysis of concentration response curves using SPSS 16.0. The statistical significance between two groups was determined using two-way ANOVA followed by Student's *t*-test. The *p*-values less than 0.05 were considered statistically significant.

## Results

### Generation of anti-DR5 ADC, Zapadcine-1

**Figure 1:**
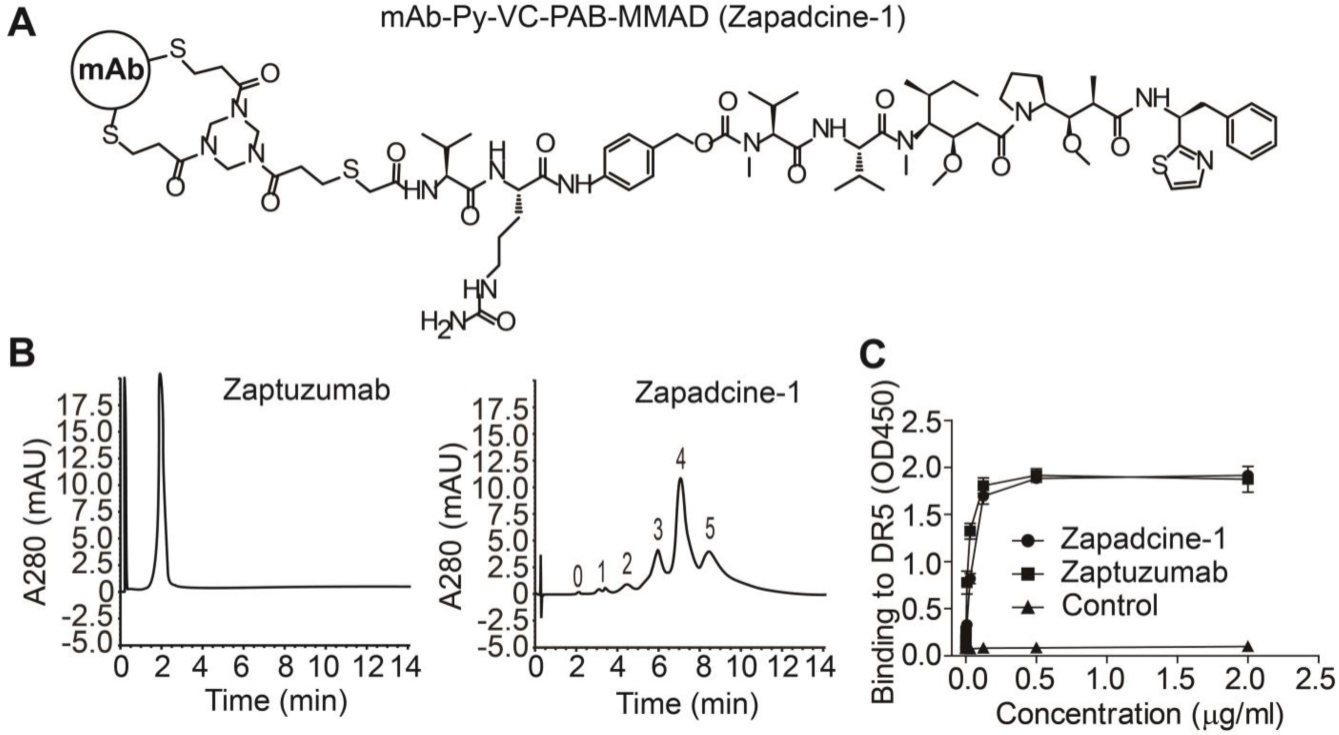
** Characterization of Zapadcine-1. (A) Molecular structures of Zapadcine-1.** Conjugates were prepared by controlled partial reduction of internal Zaptuzumab disulfides with tris (2-carboxyethyl) phosphine hydrochloride (TCEP), followed by adding various linker-drugs. Stable thioether-linked ADC was formed by the reaction of maleimides in the drug molecules with free sulfhydryl groups in the monoclonal antibodies (mAbs). (B) Hydrophobic interaction chromatography (HIC) analysis of Zapadcine-1 in a butyl-NPR column yielded five predominant peaks corresponding to the ADCs containing zero, one, two, three, four and five toxin molecules. (C) ELISA assay for the binding of Zapadcine-1 with DR5 on the cell surfaces. Naked humanized antibody Zaptuzumab was used as control.

At first, we synthesized a series of ADCs with the humanized anti-DR5 antibody Zaptuzumab coupled with toxins including MMAD, MMAE, MMAF, DM1, and DM4 etc. via various linkers. Bioactivity screening assay showed that Zapadcine-1 conjugated with MMAD via the cleavable linker PY-Val-Cit-PAB possesses the highest tumoricidal activity (unpublished data). Zapadcine-1 is composed of an anti-DR5 humanized antibody (Zaptuzumab) coupled with a cleavable valine-citrulline-dipeptide linker (PY-Val-Cit-PAB) 32 and a highly potent microtubule-disrupting toxin, monomethyl auristatin D (MMAD) by ThiolBridge technology (Figure 1A). The anti-DR5 antibody contains 4 pairs of interchain disulfide bonds, which could be reduced with dithiothreitol, tris (2-carboxyethyl) phosphine or other mild reducing agents, and resulted in eight sulfhydryl groups. By using ThioBridge technology 32, each two sulfhydryl groups covalently linked with one linker-toxin molecule, therefore, Zapadcine-1 with DAR (drug-antibody ratio) equal to 4 are mostly produced, however, ADCs with DAR less or more than 4 could be also randomly produced due to the single disulfide bond couples with one linker-toxin molecule during the preparation process (Figure 1B).

The binding activity determined by ELISA demonstrated that Zapadcine-1 possesses a similar affinity to the naked antibody Zaptuzumab (Figure 1C). Stability assay of Zapadcine-1 in human plasma showed that 0.2 to 200 μg/mL of Zapadcine-1 incubated at 37 °C in human plasma for 7 days, the free form of MMAD detected by LC/MS/MS is extremely low, indicating that Zapadcine-1 in human plasma at physiological temperature, pH and ionic strength is very stable (Figure S1).

### Zapadcine-1 binds with DR5^+^ tumor cells specifically and can be endocytosed into lysosome rapidly

**Figure 2:**
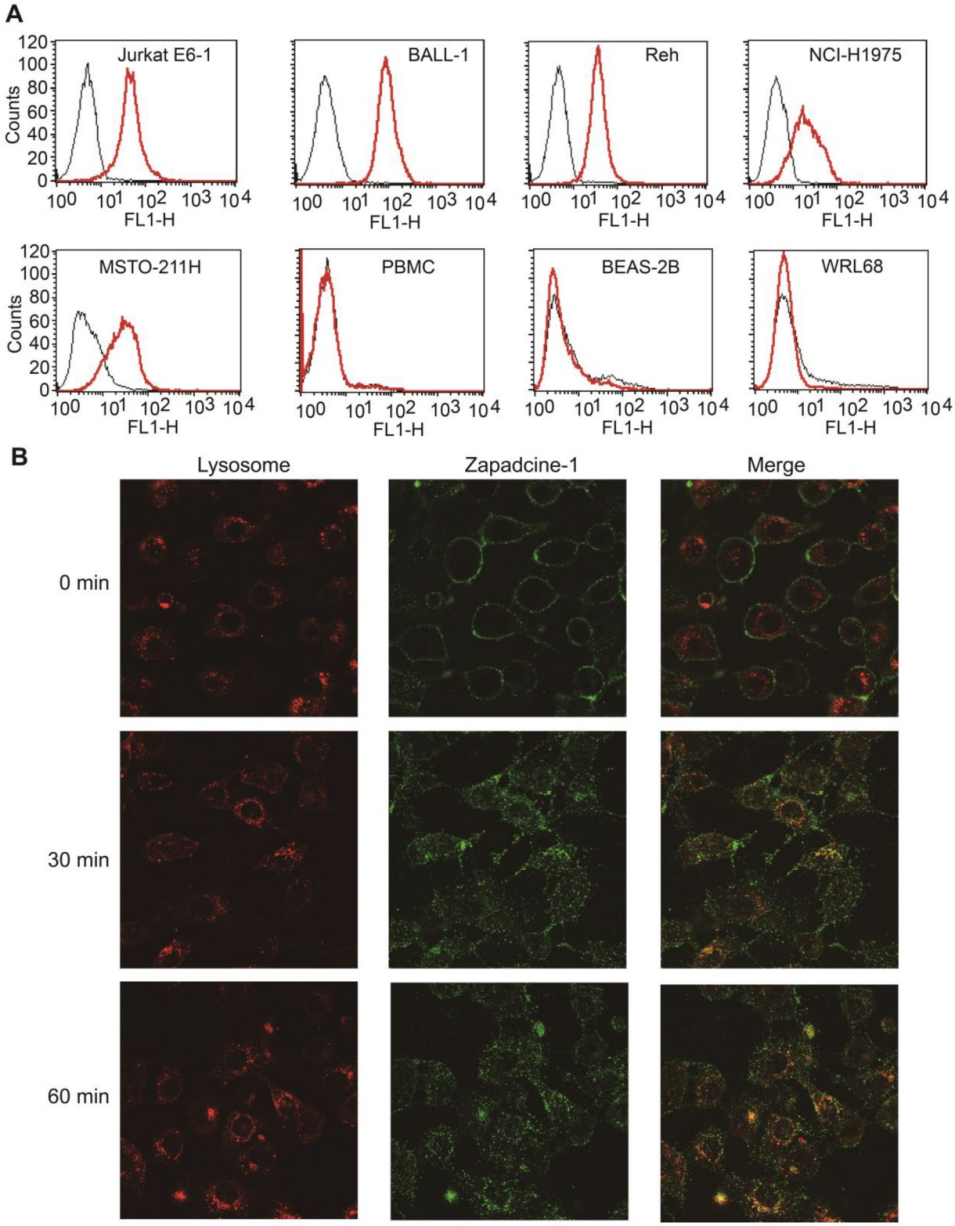
** Binding specificity and endocytosis assay of Zapadcine-1. (A).** Jurkat E6-1, J.gamma1, A3, BALL-1, THP-1, Reh of acute lymphocyte leukemia cells and NCI-H1975 and MSTO-211H lung cancer cells were incubated with Zapadcine-1 or anti-human IgG antibody. Cell-associated fluorescence was determined by FACS. **(B)** Approximately 5×10^4^ cells of MSTO-211H were incubated with 20 µg/mL of FITC-labeled Zapadcine-1 (green) and 1.0 mL of lysosome specific dye of Lyso-Tracker Red. Images were captured by the laser scanning confocal microscopy.

Binding specificity assay showed that Zapadcine-1 specifically binds to DR5^+^ tumor cells of Jurkat E6-1, BALL-1 and Reh lymphocyte leukemia cells, as well as NCI-H1975 and MSTO-211H lung cancer cells, but not normal cells of PBMC, BEAS-2B and WRL68 tested (Figure 2A).

ADC internalization is one of the key requirements for its drugability. To this end, endocytosis assay for Zapadcine-1 was carried out and showed that FITC-labeled Zapadcine-1 (green) on DR5-expressing MSTO-211H cells could be internalized rapidly into the lysosome tracking with lysosome specific Lyso-Tracker red dye (Figure 2B), suggesting that lysozyme proteases, such as tissue protease B (cathepsin B) and plasma enzyme (plasmin), could be able to break the amide bond between Val-Cit and aniline in the linker of Zapadcine-1, and therefore result in the self-elimination of the p-aminobenzyl carbonyl to release MMAD killing tumor cells 37, 38.

### Cytotoxicity of Zapadcine-1 in tumor cells

**Figure 3:**
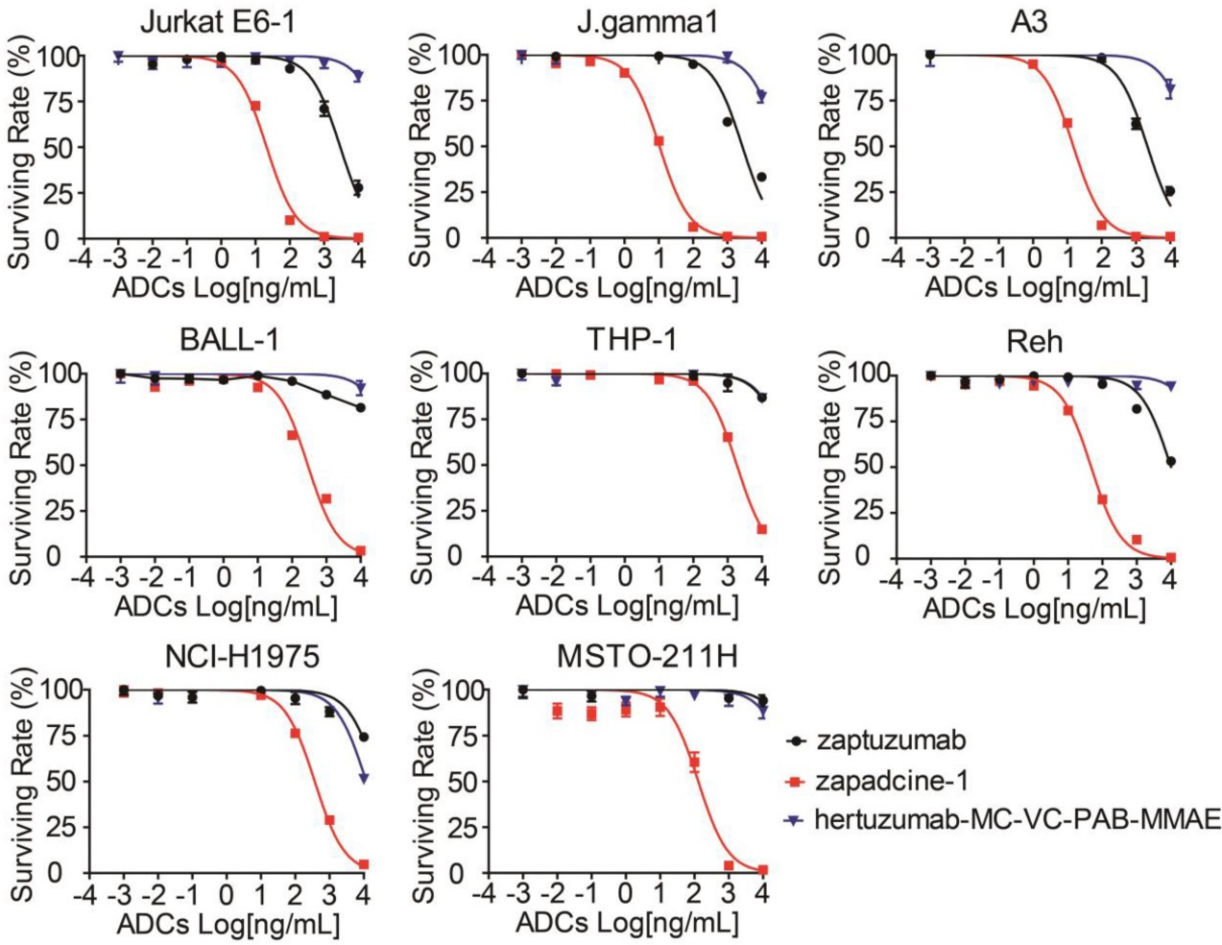
***In vitro* cytotoxicity of Zapadcine-1 to the DR5-expressing tumor cells.** Jurkat E6-1, J.gamma1, A3, BALL-1, THP-1, Reh, NCI-H1975 and MSTO-211H cells were incubated with the indicated concentrations of Zapadcine-1, Zaptuzumab, non-targeting ADC (hertuzumab-MC-VC-PAB-MMAE), respectively. The cytotoxicity of Zapadcine-1 was determined by CellTiter-Glo® Luminescent Cell Viability Assay according to the manufacturer's instructions. Absorbance at 450 nm was measured on SPARK 10M Multiplate Reader. The cell survival was calculated using the following formula: A_sample_/A_control_×100%. The IC50 was calculated by SPSS software. Data are presented as mean ± SD.

To investigate the cytotoxicity of Zapadcine-1, various human tumor cells and normal cells at their logarithm phases were collected and treated for 48 h with Zapadcine-1 at the concentrations of 0.00, 0.01, 0.10, 1.00, 10.00, 100.00, 1,000.00, 10,000.00 ng/mL. Cell viability assay showed that Zapadcine-1 possesses a strong cytotoxicity in Jurkat E6-1, J.gamma1, A3, BALL-1, THP-1 and Reh acute lymphocyte leukemia cells with IC50s of 20.29 ± 0.25 ng/mL, 10.52 ± 0.09 ng/mL, 14.63 ± 0.12 ng/mL, 285.6 ± 11.11 ng/mL, 1874 ± 28.15 ng/mL and 46.68 ± 0.52 ng/mL, respectively, and in NCI-H1975 and MSTO-211H lung cancer cells with IC50 of 373.1 ± 5.09 ng/mL and 126.5 ± 18.84 ng/mL, respectively. The cytotoxicity of Zapadcine-1 to all the tumor cells tested is much stronger than the controls of naked monoclonal antibody Zaptuzumab and the non-targeted ADC, anti-HER-2 ADC of hertuzumab-MC-VC-PAB-MMAE (Figure 3). However, 12 human normal cell lines including PBMC, BEAS-2B, WRL68, CCD-18Co, HFL1, NCM-460, SV-HUC-1, HSF, hFOB 1.19, HBL-100, Hs578Bst and MRC-5 are all resistant to Zapdcine-1 cytotoxicity (Figure S2). These data show that Zapadcine-1 specifically kills tumor cells dose-dependently, but not normal cell lines tested.

### Efficacy of Zapadcine-1 for cancer therapy in animal models

**Figure 4:**
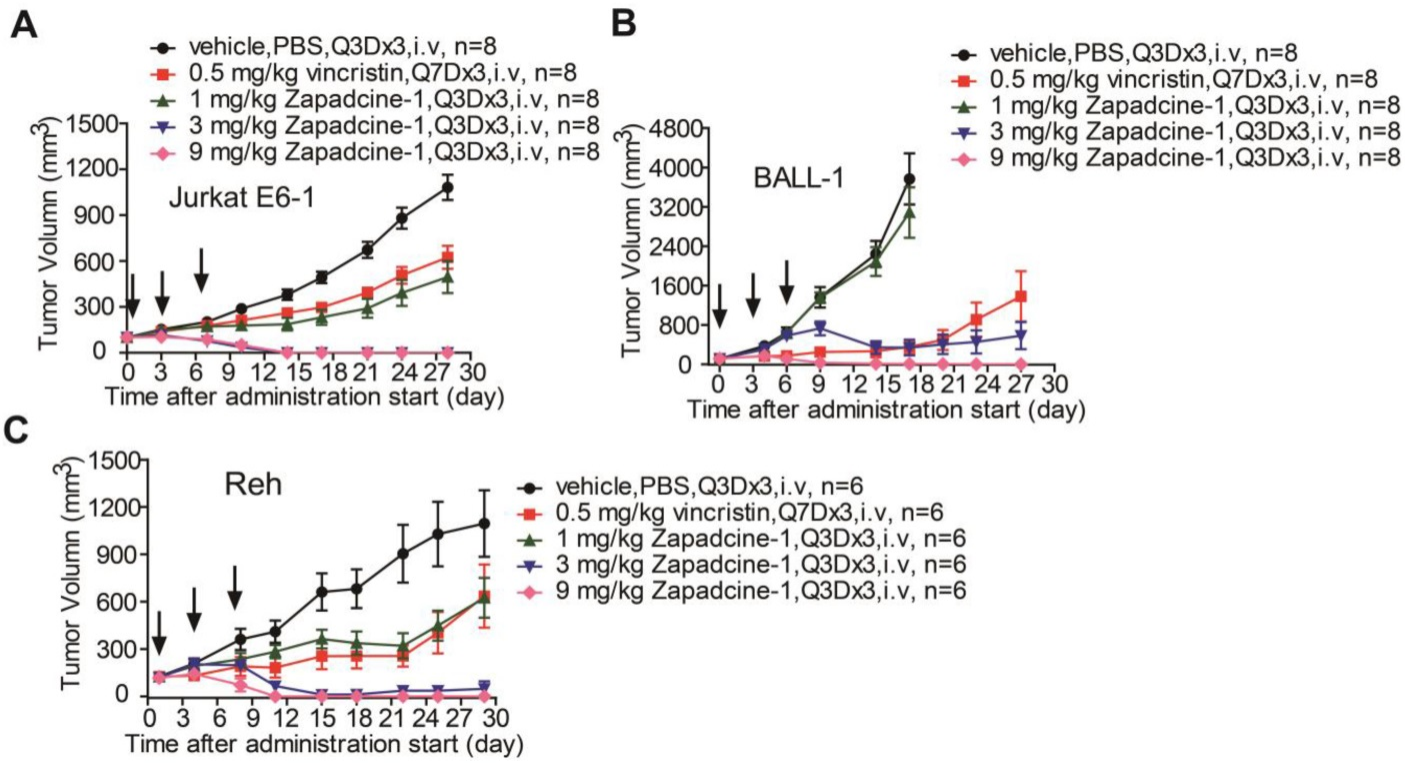
***In vivo* efficacy of Zapadcine-1 in acute lymphocyte leukemia mouse CDX models**. NOD/SCID mice were injected s.c. with 1×10^7^ acute lymphocyte leukemia cells Jurkat E6-1 (A), BALL-1 (B) and Reh (C), respectively. Mice bearing xenografts (tumor size approximately averaged 100-200 mm^3^) intravenously received saline, 1.0 mg/kg, 3.0 mg/kg and 9.0 mg/kg of Zapadcine-1, or 0.5 mg/kg of vincristine at day 0, day 4 and day 7. Tumor sizes were measured twice a week, and tumor volumes were determined according to the formula: tumor volume (mm^3^) = longer diameter × (shorter diameter)^2^ × 0.5. The inhibition rate of tumor growth was calculated as [1 - (tumor volume treated final - tumor volume treated initial)/(tumor volume control final - tumor volume control initial)] × 100%. Q3D×3, every three days for 3 times.

Subsequently, efficacy of Zapadcine-1 suppressing tumor growth was evaluated in animal models. NOD-SCID or BALB/c nude mouse subcutaneous xenografts of human acute lymphocyte leukemia cells, Jurkat E6-1, BALL-1 and Reh were established, and the tumors were allowed to develop until the average volumes reached at least 100 mm^3^. The tumor-bearing mice were then randomly divided into groups. The animals were administered intravenously via tail vein with vehicle (PBS, Q3D×3), vincristine (0.5 mg/kg, Q7D×3), and Zapadcine-1 (1.0 mg/kg, 3.0 mg/kg and 9.0 mg/kg, Q3D×3), respectively. As shown in Figure 4, within 21 days after administration start, the tumor volumes in Zapadcine-1 treated groups were strongly shrunk, especially the tumors were completely removed in Zapadcine-1 of 3.0 mg/kg and 9.0 mg/kg groups on day 12 after administration, comparing with the untreated and vincristine treated groups. The tumor weights at the end of experiments confirmed these results (Figure S3). In addition, histopathological images of the mouse dissected organs showed that Zapadcine-1 did not have notable toxic side effects on heart, liver, lung and kidney (Figure S4). These data clearly indicate that Zapadcine-1 possesses an excellent therapeutic efficacy in the three types of acute lymphocyte leukemia.

**Figure 5:**
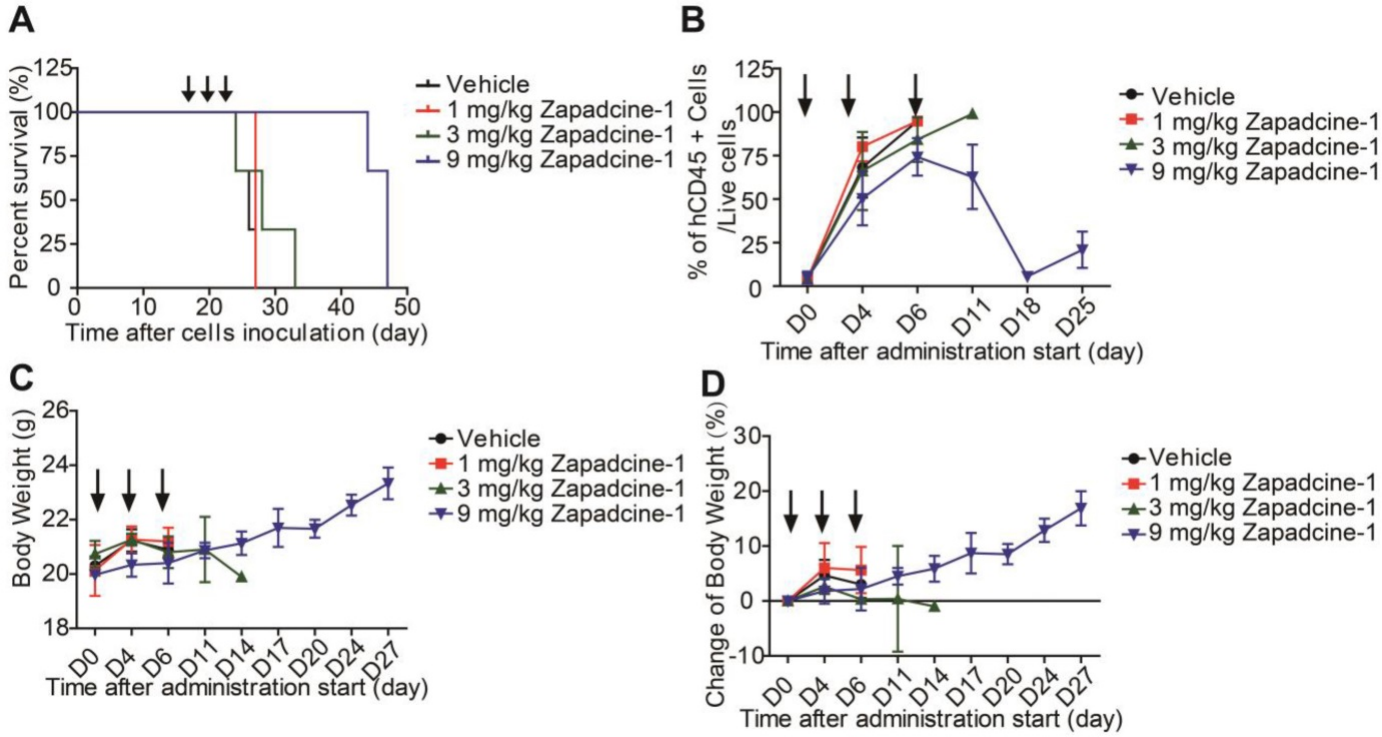
** Evaluation of the *in vivo* efficacy of Zapadcine-1 in HuKemia® acute lymphocyte leukemia PDX model AL7442.** 8×10^5^ patient AL7442 cells were intravenously injected into NOD/SCID mice for tumor development. After inoculation, blood sample were weekly collected for staining human CD45^+^ cells in mouse PBMC and analyzed by FACS to monitor the tumor burden. When average tumor burden reached about 4%, mice were randomly allocated into 4 groups (n = 3). The mice were intravenously injected with saline, 1.0 mg/kg, 3.0 mg/kg and 9.0 mg/kg of Zapadcine-1 on day 0, day 4 and day 7, respectively. **(A)** *In vivo* anti-tumor activity of Zapadcine-1 in HuKemia® Acute Leukemia PDX Model AL7442. Kaplan-Meier survival plots show percentage animal survival over 46 days, in which Zapadcine-1 was administered 9.0 mg/kg in comparison with vehicle. **(B)** Tumor burden of AL7442 mice. **(C and D)** Body weight and change of AL7442 mice. Q3D×3, every three days for 3 times.

To further confirm the efficacy of Zapdcine-1 in acute lymphocyte leukemia, T lymphocyte leukemia of PDX model was established in NOD-SCID mice by using HuKemia^®^ AL7442 patient lymphocyte leukemia cells. Zapadcine-1 was injected through tail vein of the mice (1.0 mg/kg, 3.0 mg/kg and 9.0 mg/kg, Q3D×3). The ratio of human CD45^+^ cells to the live cells in the mouse peripheral blood as mouse tumor burden index was weekly detected. Observation of the animal survival showed that the mice injected with 9.0 mg/kg of Zapadcine-1 survived for 27 days, but the mice injected with vehicle, 1.0 mg/kg and 3.0 mg/kg of Zapadcine-1 survived for 7 to 9 days after administration (Figure 5A). Accordingly, the ratios of CD45^+^ cells to the live cells were rapidly decreased to almost zero from day 6 to 18 after three injections, but not in the groups injected with 1.0 mg/kg, 3.0 mg/kg of Zapadcine-1 and the vehicle control (Figure 5B). Importantly, there was no visible influence on the body weights and body weight changes in the animals administrated with Zapadcine-1 (Figure 5C and 5D). These data demonstrate that Zapadcine-1 significantly and dose-dependently suppresses tumor growth in acute lymphocyte leukemia with acceptable safety profile.

**Figure 6:**
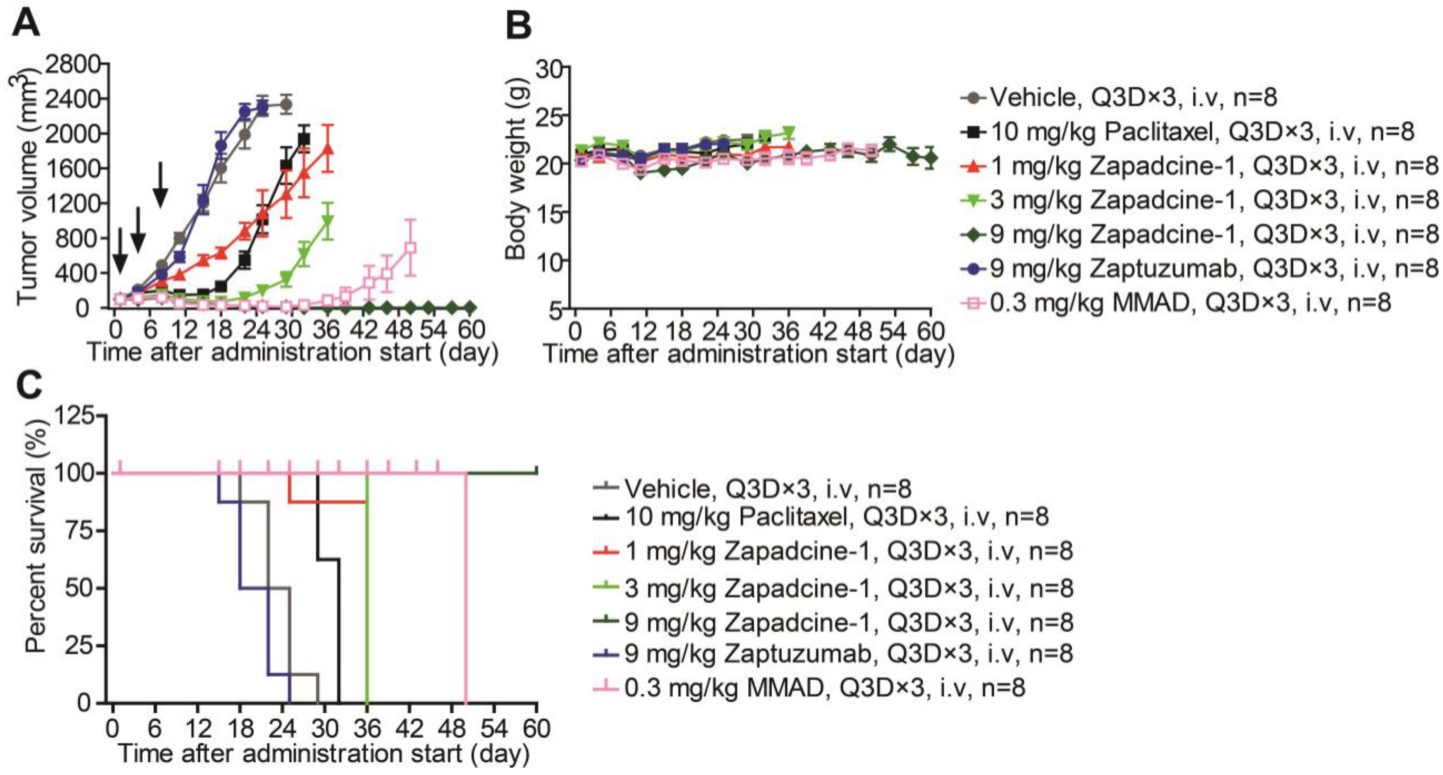
***In vivo* efficacy of Zapadcine-1 in NSCLC NCI-H1975 mouse CDX model.** BALB/c mice were injected s.c. with 1×10^5^ NCI-H1975 cells. Mice bearing xenografts (tumor size approximately averaged 100 - 200 mm^3^) intravenously received saline, 1.0 mg/kg, 3.0 mg/kg and 9.0 mg/kg of Zapadcine-1, 10.0 mg/kg of control Zaptuzumab, 0.3 mg/kg of MMAD, or 10.0 mg/kg of paclitaxel on day 0, day 4 and day 7 (n = 8). Tumor volumes (A), body weights (B) and animal survival (C) were observed. Q3D×3, every three days for 3 times.

The therapeutic efficacy of Zapadcine-1 for cancers was further checked in human lung cancer (NSCLC) mouse subcutaneous xenografts established by using NCI-H1975 cells. While tumor volumes were reached at least 100 mm^3^, the tumor-bearing mice were randomly divided into groups. The mice were administered intravenously via tail vein injection with Zapadcine-1 (1.0 mg/kg, 3.0 mg/kg and 9.0 mg/kg, Q3D×3), respectively. The vehicle (PBS), paclitaxel (10.0 mg/kg), Zaptuzumab (9.0 mg/kg), and MMAD (0.3 mg/kg) were used as controls (Q3D×3). As shown in Figure 6A, the tumor growth in all therapeutic groups with Zapadcine-1 were markedly suppressed after three injection, especially the tumors in 9 mg/kg group completely regressed, comparing with all the controls. The therapeutic efficacy without tumor relapses remained till 60 days when the experiments were ended. Similarly, all the treatments did not lead to a notable change of mouse body weights (Figure 6B and 6C). These data indicate that Zapadcine-1 is also potential for lung cancer therapy.

### Toxicity of Zapadcine-1 in rat and cynomolgus monkey

**Figure 7:**
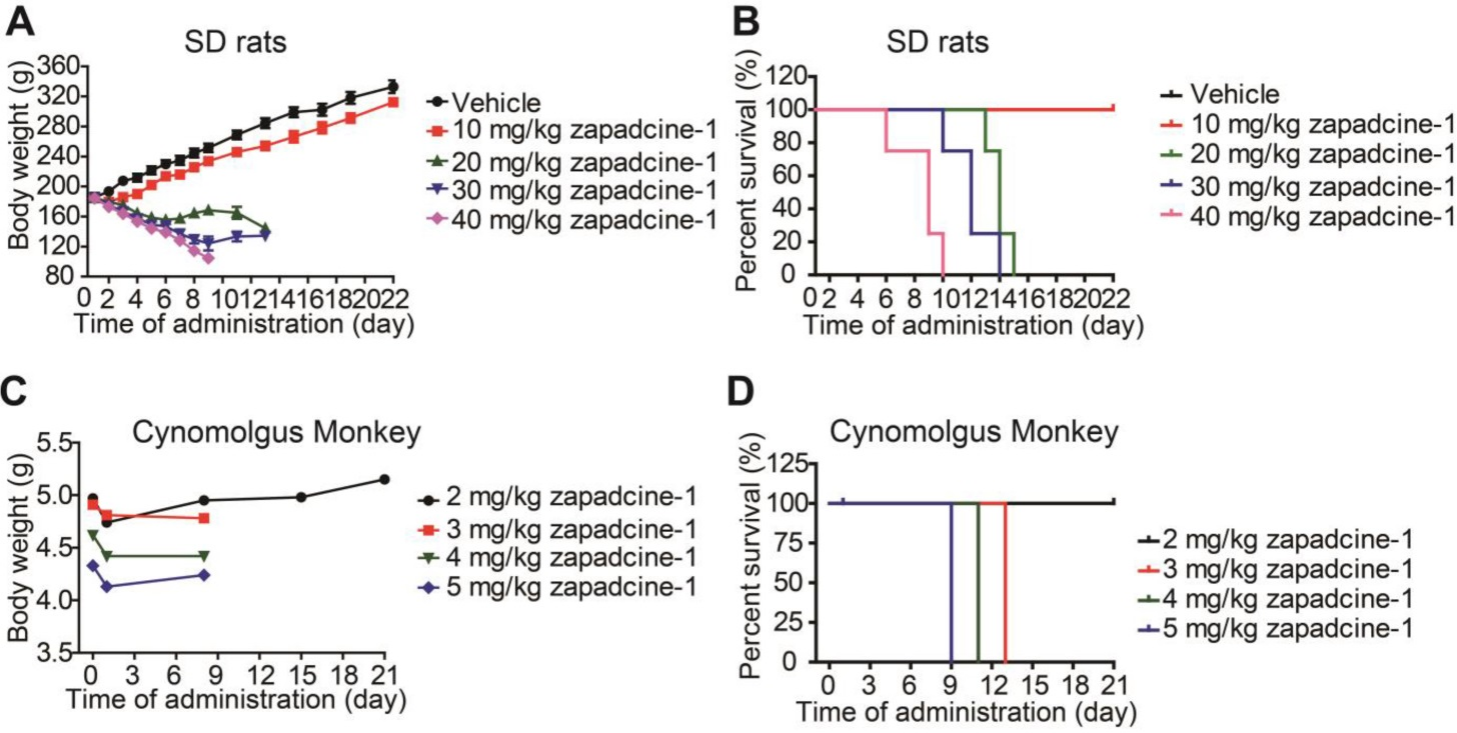
***In vivo* toxicity of Zapadcine-1.** MTD assay was conducted in male SD rats and cynomolgus monkey. The rats were single-dosed intravenously on days 1 with vehicle, 10 mg/kg, 20 mg/kg, 30 mg/kg or 40 mg/kg of Zapadcine-1 (n = 4). Body weight of SD rats (A) and survival rate (B) were observed. Kaplan-Meier survival plots show the percentage of rat survival over day 21. The cynomolgus monkeys were single-dosed intravenously on day 1 with vehicle, 2.0 mg/kg, 3.0 mg/kg, 4.0 mg/kg or 5.0 mg/kg of Zapadcine-1 (n = 1). (C) Body weight of cynomolgus monkey. Kaplan-Meier survival plots (D) show the percentage of animal survival over day 21.

As mentioned above all the mice administrated with Zapadcine-1 in mouse xenograft models were appeared healthy and no notable body weight changes observed. Dorywalska et al. reported that MMAD metabolism in mouse plasma is less stable than rat, monkey and human 25. Therefore, it is necessary to check the toxicity of Zapadcine-1 in SD rats. To do so, a single dose of Zapadcine-1 (10 mg/kg to 40 mg/kg) was intravenously injected into SD rats. The general appearances and body weights were monitored for three weeks. As shown in Figure 7A, Zapadcine-1 up to 10 mg/kg were well tolerated without apparent toxicity, all the rats showed normal growth, good exercise, normal body weight, normal liver and kidney function. In 20 mg/kg injection group, the animals experienced weight loss, and all the animals were died on day 15. In 30 mg/kg injection group, the animals experienced weight loss, and all the animals were died at day 13. All the animals in the group of 40 mg/kg injection were died at day 10 (Figure 7B). Thus, the maximal toxic dose (MTD) of Zapadcine-1 in SD rat was determined to be 10 mg/kg to 20 mg/kg.

We further demonstrated that both Zaptuzumab and Zapadcine-1 could bind with the sections of heart, liver and kidney of cynomolgus monkey (Figure S5). The safety of Zapadcine-1 in cynomolgus monkey was also detected. A single dose of Zapadcine-1 (2.0 mg/kg, 3.0 mg/kg, 4.0 mg/kg and 5.0 mg/kg) was intravenously administered into cynomolgus monkeys, respectively. The general appearances and body weights were monitored for three weeks. As shown in Figure 7C and 7D, the monkeys injected with 2.0 mg/kg of Zapadcine-1 was well tolerated with no adverse effects on body weights and clinical toxic indexes, such as erythrocytes, hemoglobin and hematocrit, and well survived till to the end of experiments with no cardiovascular, respiratory, renal, gastrointestinal, neurologic, and ophthalmic abnormalities observed. The monkeys in 3 mg/kg and 4 mg/kg groups showed symptoms such as reduced activity, loss of appetite, arched back posture, and nasal secretions at day 10 and died at day 13 and 11, respectively (Figure S6, Table S1). The monkeys in 5 mg/kg group showed loss of appetite, reduced activity, arched back posture, gingival discoloration, skin discoloration and cold skin when touching, and died at day 9 after administration. Thus the MTD of Zapadcine-1 in monkey is 2 mg/kg to 3 mg/kg.

In summary, these data demonstrate that the anti-DR5 ADC, Zapadcine-1, is a very promising candidate for further exploration of its potential as a novel cancer therapeutic agent, especially for the acute lymphocyte leukemia.

## Discussion

It is well known that DR4/DR5 are highly expressed in malignant cells, including leukemia, non-small cell lung carcinoma, pancreatic cancer, colon cancer, breast cancer, ovarian cancer and bladder cancer, but less or no expression in the most normal tissues and cells 13, 27, 39, 40. Cancer therapeutics targeting DR4/DR5 including the first generation of rsTRAIL and anti-DR4/DR5 agonistic monoclonal antibodies have disappointed in the clinical trials 16, 41. Recently, the second generation of TRAIL agonists, such as rsTRAIL fused to Fc, single chain of monoclonal antibodies targeting DR4/DR5 functionalized to nanoparticles or linkers, in order to increase their agonistic activity and efficacy for cancer therapy, have been developed in the preclinical or early clinical trials 8, 18, 19, indicating that the second generation of TRAIL receptor agonists or their derivatives may raise up a new hope for TRAIL receptor targeting cancer therapy. In the present study we have demonstrated that the anti-DR5 antibody-drug conjugate (ADC), Zapadcine-1, could be one of such excellent agents for acute lymphocyte leukemia and solid tumor therapy.

At first, we synthesized a series of ADCs with the humanized anti-DR5 antibody Zaptuzumab coupled with toxins including MMAD, MMAE, MMAF, DM1, and DM4 via various linkers. Bioactivity screening assay showed that Zapadcine-1 conjugated with MMAD via the cleavable linker PY-Val-Cit-PAB possesses the highest tumoricidal activity. Then we demonstrated that Zapadcine-1 binds DR5 with a comparable affinity as the naked antibody Zaptuzumab, displaying that the conjugation of antibody with linker PY-Val-Cit-PAB and MMAD does not affect the affinity for its antigen. Further experiments showed that Zapadcine-1 binds with Jurkat E6-1, BALL-1 and Reh acute lymphocyte leukemia cells as well as NCI-H1975 and MSTO-211H non-small cell lung cancer cells, but not or less binds with the normal cells of PBMC, BEAS-2B and WRL68 tested, proving that Zapadcine-1 targets to the tumor cells specifically. Accordingly, cytotoxicity assay showed that Zapadcine-1 powerfully kills DR5 positive tumor cells, but not normal cells tested. *In vivo* efficacy study showed that Zapadcine-1 at 1.0 mg/kg, 3.0 mg/kg and 9 mg/kg suppressed human acute T lymphocyte leukemia mouse xenografts in both CDX and PDX models, especially in the 3.0 mg/kg and 9.0 mg/kg groups, Zapadcine-1 cleaned up all the tumor cells in Jurkat E6-1 and Reh CDX models. Similarly, in NCI-H1975 CDX models, Zapadcine-1 displayed a dose-dependent efficacy in anti-tumor activity in tumor-baring mice. It is importantly Zapadcine-1 at 9.0 mg/kg induces a complete, durable tumor disappearance in all the animals from day 17 to day 60 after administration start, further illustrating a markedly dose-dependent suppression in tumor growth and elongation of the tumor-free survival. Comparison with MMAD single drug control at a comparable dosage of 0.3 mg/kg although induces a complete tumor regression in the animals from day 18 to day 27 after administration start, the tumors were relapsed and grew rapidly afterwards, indicating that Zapadcine-1 can get rid of the tumors at 9.0 mg/kg in mice. Preliminary safety evaluation demonstrated that single dose injection showed that the MTD of Zapadcine-1 was up to 10 mg/kg to 20 mg/kg in SD rats and 2.0 mg/kg to 3.0 mg/kg in cynomolgus monkey, respectively. Thinking about the therapeutic dosages in mouse CDX and PDX model were as lower as 3 mg/kg to 9 mg/kg, which is approximately equal to less than 0.3 mg/kg to 0.9 mg/kg in human, the MTD of 2.0 mg/kg to 3.0 mg/kg in monkey is acceptable, and the therapeutic window of Zapadcine-1 is excellent for human cancer therapy.

In summary, we have generated and developed a novel antibody-drug conjugate, Zapadcine-1, as a potential therapeutic for DR5 positive malignancies. Since Zapadcine-1 can be effectively internalized into the lysosome and quickly release the toxin MMAD in the cells, therefore to kill DR5 positive tumor cells specifically, including acute T lymphocyte leukemia, acute B lymphocyte leukemia, acute Reh lymphocyte leukemia (non-T and non-B), non-small cell lung cancers. No DR5 negative normal cells could be casualty. Furthermore, while the anti-DR5 ADC kills cancer cells by intracellular released payload, DR5-mediated complex signaling pathways are not stirred up, therefore, DR5 expression could be a unique molecular marker for the selection of patients, who may be advantaged from the anti-DR5 ADC therapy. Taken together, we demonstrated that anti-DR5 antibody-drug conjugates possesses a promising preclinical cytotoxic activity for DR5 positive cancers, meriting further exploration of its potential as a novel cancer therapeutics especially for the acute lymphocyte leukemia therapy.

## Supplementary Material

Supplementary figures and tables.

## References

[1] Levine B, Deretic V (2007). Unveiling the roles of autophagy in innate and adaptive immunity. Nat Rev Immunol.

[2] Eskelinen EL, Saftig P (2009). Autophagy: a lysosomal degradation pathway with a central role in health and disease. Biochim Biophys Acta.

[3] Ciechanover A (2013). Intracellular protein degradation: from a vague idea through the lysosome and the ubiquitin-proteasome system and onto human diseases and drug targeting. Bioorg Med Chem.

[4] Ramos-Medina R, Montes-Moreno S, Maestre L, Canamero M, Rodriguez-Pinilla M, Lazaro A (2011). Immunohistochemical analysis of HLDA9 workshop antibodies against cell-surface molecules in reactive and neoplastic lymphoid tissues. Immunol Lett.

[5] Geng C, Hou J, Zhao Y, Ke X, Wang Z, Qiu L (2014). A multicenter, open-label phase II study of recombinant CPT (Circularly Permuted TRAIL) plus thalidomide in patients with relapsed and refractory multiple myeloma. Am J Hematol.

[6] Pan G, Ni J, Wei YF, Yu G, Gentz R, Dixit VM (1997). An antagonist decoy receptor and a death domain-containing receptor for TRAIL. Science.

[7] Pan G, O'Rourke K, Chinnaiyan AM, Gentz R, Ebner R, Ni J (1997). The receptor for the cytotoxic ligand TRAIL. Science.

[8] Agathe Dubuisson OM (2017). Antibodies and derivatives targeting DR4 and DR5 for cancer therapy. Antibodies.

[9] Walczak H, Degli-Esposti MA, Johnson RS, Smolak PJ, Waugh JY, Boiani N (1997). TRAIL-R2: a novel apoptosis-mediating receptor for TRAIL. EMBO J.

[10] Gores GJ, Kaufmann SH (2001). Is TRAIL hepatotoxic?. Hepatology.

[11] Koschny R, Walczak H, Ganten TM (2007). The promise of TRAIL-potential and risks of a novel anticancer therapy. J Mol Med (Berl).

[12] Lawrence D, Shahrokh Z, Marsters S, Achilles K, Shih D, Mounho B (2001). Differential hepatocyte toxicity of recombinant Apo2L/TRAIL versions. Nat Med.

[13] Chen L, Qiu Y, Hao Z, Cai J, Zhang S, Liu Y (2017). A novel humanized anti-tumor necrosis factor-related apoptosis-inducing ligand-R2 monoclonal antibody induces apoptotic and autophagic cell death. IUBMB Life.

[14] Lv F, Qiu Y, Zhang Y, Liu S, Shi J, Liu Y (2011). Adeno-associated virus-mediated anti-DR5 chimeric antibody expression suppresses human tumor growth in nude mice. Cancer Lett.

[15] El-Gazzar A, Perco P, Eckelhart E, Anees M, Sexl V, Mayer B (2010). Natural immunity enhances the activity of a DR5 agonistic antibody and carboplatin in the treatment of ovarian cancer. Mol Cancer Ther.

[16] Naoum GE, Buchsbaum DJ, Tawadros F, Farooqi A, Arafat WO (2017). Journey of TRAIL from bench to bedside and its potential role in immuno-oncology. Oncol Rev.

[17] Del Mistro G, Lucarelli P, Muller I, De Landtsheer S, Zinoveva A, Hutt M (2018). Systemic network analysis identifies XIAP and IκBα as potential drug targets in TRAIL resistant BRAF mutated melanoma. NPJ Syst Biol Appl.

[18] Li L, Wen XZ, Bu ZD, Cheng XJ, Xing XF, Wang XH (2016). Paclitaxel enhances tumoricidal potential of TRAIL via inhibition of MAPK in resistant gastric cancer cells. Oncol Rep.

[19] Jaworska D, Szliszka E (2017). Targeting apoptotic activity against prostate cancer stem cells.

[20] Lambert JM, Morris CQ (2017). Antibody-drug conjugates (ADCs) for personalized treatment of solid tumors: A review. Adv Ther.

[21] Beck A, Goetsch L, Dumontet C, Corvaia N (2017). Strategies and challenges for the next generation of antibody-drug conjugates. Nat Rev Drug Discov.

[22] Dholaria B, Hammond W, Shreders A, Lou Y (2016). Emerging therapeutic agents for lung cancer. J Hematol Oncol.

[23] Panowski S, Bhakta S, Raab H, Polakis P, Junutula JR (2014). Site-specific antibody drug conjugates for cancer therapy. MAbs.

[24] Sievers EL, Senter PD (2013). Antibody-drug conjugates in cancer therapy. Annu Rev Med.

[25] Dorywalska M, Strop P, Melton-Witt JA, Hasa-Moreno A, Farias SE, Galindo Casas M (2015). Site-dependent degradation of a non-cleavable auristatin-based linker-payload in rodent plasma and its effect on ADC efficacy. PloS One.

[26] Jain N, Smith SW, Ghone S, Tomczuk B (2015). Current ADC linker chemistry. Pharm Res.

[27] Guo Y, Chen C, Zheng Y, Zhang J, Tao X, Liu S (2005). A novel anti-human DR5 monoclonal antibody with tumoricidal activity induces caspase-dependent and caspase-independent cell death. J Biol Chem.

[28] Diao Z, Shi J, Zhu J, Yuan H, Ru Q, Liu S (2013). TRAIL suppresses tumor growth in mice by inducing tumor-infiltrating CD4(+)CD25(+) Treg apoptosis. Cancer Immunol Immunother.

[29] Li M, Wu Y, Qiu Y, Yao Z, Liu S, Liu Y (2012). 2A peptide-based, lentivirus-mediated anti-death receptor 5 chimeric antibody expression prevents tumor growth in nude mice. Mol Ther.

[30] Shi J, Liu Y, Zheng Y, Guo Y, Zhang J, Cheung PT (2006). Therapeutic expression of an anti-death receptor 5 single-chain fixed-variable region prevents tumor growth in mice. Cancer Res.

[31] Tang W, Wang W, Zhang Y, Liu S, Liu Y, Zheng D (2009). TRAIL receptor mediates inflammatory cytokine release in an NF-kappaB-dependent manner. Cell Res.

[32] Huang C, Fang J, Ye H, Zhang L (2018). Covalent linkers in antibody-drug conjugates and methods of making and using the same.

[33] Yao X, Jiang J, Wang X, Huang C, Li D, Xie K (2015). A novel humanized anti-HER2 antibody conjugated with MMAE exerts potent anti-tumor activity. Breast Cancer Res Treat.

[34] Hamblett KJ, Senter PD, Chace DF, Sun MM, Lenox J, Cerveny CG (2004). Effects of drug loading on the antitumor activity of a monoclonal antibody drug conjugate. Clin Cancer Res.

[35] Nair AB, Jacob S (2016). A simple practice guide for dose conversion between animals and human. J Basic Clin Pharm.

[36] Senter PD (2009). Potent antibody drug conjugates for cancer therapy. Curr Opin Chem Biol.

[37] Okeley NM, Miyamoto JB, Zhang X, Sanderson RJ, Benjamin DR, Sievers EL (2010). Intracellular activation of SGN-35, a potent anti-CD30 antibody-drug conjugate. Clin Cancer Res.

[38] Francisco JA, Cerveny CG, Meyer DL, Mixan BJ, Klussman K, Chace DF (2003). cAC10-vcMMAE, an anti-CD30-monomethyl auristatin E conjugate with potent and selective antitumor activity. Blood.

[39] Qiu Y, Zhang Z, Shi J, Liu S, Liu Y, Zheng D (2012). A novel anti-DR5 chimeric antibody and epirubicin synergistically suppress tumor growth. IUBMB Life.

[40] Zhang P, Zheng Y, Shi J, Zhang Y, Liu S, Liu Y (2010). Targeting a novel N-terminal epitope of death receptor 5 triggers tumor cell death. J Biol Chem.

[41] Shlyakhtina Y, Pavet V, Gronemeyer H (2017). Dual role of DR5 in death and survival signaling leads to TRAIL resistance in cancer cells. Cell Death Dis.

