# Supplementary material for: A novel anti-DR5 antibody-drug conjugate possesses a high-potential therapeutic efficacy for leukemia and solid tumors
Source: Theranostics. 2019 Jul 13;9(18):5412–23. doi: 10.7150/thno.33598 (PMC6691585; doi:10.7150/thno.33598)
Supplement: Supplementary file 1 — Supplementary figures and tables. [file thnov09p5412s1.pdf]

Supported Information

A novel anti-DR5 antibody-drug conjugate possesses a high-potential therapeutic efficacy for leukemia and solid tumors

Shuyong Zhang, Chao Zheng, Wan Zhu, Peng Xiong, Dongdong Zhou, Changjiang Huang, and Dexian Zheng

Supplementary Materials and Methods

In vitro cytotoxicity assay of human normal cells

Human normal peripheral blood mononuclear cells (PBMC), lung epithelial cells (BEAS-2B), liver cells (WRL68), embryo lung cells (MRC-5), colon tissue cells (CCD-18Co), colon epithelial cells (NCM-460), ureteral epithelial cells (SV-HUC-1), skin fibroblasts (HSF), embryonic pulmonary fibroblasts (HFL1), osteoblasts (hFOB 1.19), mammary epithelial cells (HBL-100) and mammary gland cells (Hs578Bst) were purchased from the Cell Bank of Chinese Academy of Medical Sciences (Beijing, China), Cell Bank of Chinese Academy of Shanghai Institutes for Biological Sciences (Shanghai, China) and ATCC (American Type Culture Collection, Manassas, VA), respectively, and cultured in RPMI-1640 medium or Dulbecco's modified Eagle's medium (Gibco) supplemented with 10% fetal bovine serum (Gibco) and 1% penicillin/streptomycin (North China Pharmaceutical Co., Shijiazhuang, China) in a humidified incubator (Thermo Fisher Scientific, Waltham, MA) with 5% CO_2 at 37 ℃. The cytotoxicity of Zapadcine-1 was analyzed by the CellTiter-Glo® Luminescent Cell Viability Assay (Promega, G7572).

Zapadcine-1 stability in human plasma

Zapadcine-1 at concentrations of 0.2 to 200 μg/mL was spiked in pooled human K2-EDTA plasma and incubated at 37 °C for a time course of 1, 2, 4, 8, 24, 48, 72, 96, 120 and 144 h. The free-form MMAD in the plasma samples were determined by LC/MS/MS and calculated according to the following formula: Free MMAD (%) = actual generated MMAD concentration / total MMAD concentration ×100%, and total MMAD concentration = Zapadcine-1 theoretical molar concentration × DAR × MMAD molecular weight.

Liquid chromatography mass spectrometry (LC/MS/MS) analysis

Liquid chromatography conditions:

Column	Titank C18 1.8 μm，100×2.1 mm<br>Manufacturer：Guangzhou FLM Scientific Instrument Co. Ltd.
Flow rate	0.3 mL/min
Sampling volume	5 μL
Mobile phase	Phase A：water with 0.1% formic acid

<table>
<tr><td></td><td>Phase B：acetonitrile with 0.1% formic acid</td></tr>
<tr><td>Running time</td><td>6.2 min</td></tr>
<tr><td>Gradient</td><td>
<table>
<tr><th>Time (min)</th><th>Flow rate (mL/min)</th><th>A (%)</th><th>B (%)</th></tr>
<tr><td>0.5</td><td>0.3</td><td>75</td><td>25</td></tr>
<tr><td>3.5</td><td>0.3</td><td>40</td><td>60</td></tr>
<tr><td>3.8</td><td>0.3</td><td>40</td><td>60</td></tr>
<tr><td>3.81</td><td>0.3</td><td>5</td><td>95</td></tr>
<tr><td>5.0</td><td>0.3</td><td>5</td><td>95</td></tr>
<tr><td>5.01</td><td>0.3</td><td>75</td><td>25</td></tr>
<tr><td>6.2</td><td>Stop</td><td>75</td><td>25</td></tr>
</table>
</td></tr>
</table>

Mass spectrum conditions：

A positive ion MRM scan was used using a Trip Quad 5500 mass spectrometer with an ESI source.

Source parameters：

Ion source	CAD	CUR (psi)	Gasl (psi)	Gas2 (psi)	IS (v)	TEM (℃)
Turbo Ionspay	8	20	50	50	5500	500

Compound parameters：

Parameters / Compound	Precursor m/z	Precursor m/z	Dwell Time (ms)	DP (v)	EP (v)	CE (v)	CXP (v)
A	771.37	739.5	150	81	10	43	4
B	718.374	686.3	150	100	10	39	34

Analysis procedures:

1) Take 50 μL of the sample to be tested, add 150 μL of internal standard solution (1 ng/mL MMAD in acetonitrile with 0.1% formic acid), vortex mixing, 16000 g, and centrifuge for 10 min; draw 120 μL of supernatant, add 600 μL water with 1% formic acid and mix;

2) the above processed sample was added slowly through the solid phase extraction plate under low pressure conditions;
3) Add 200 μL of water solution with 20% methanol to each well to rinse the solid phase extraction plate, and discard the eluent;
4) Add 50 μL of eluent (acetonitrile: isopropanol: formic acid = 50:45:5) to each well to elute the solid phase extraction plate followed by another 50 μL of the eluate and collect all the eluate. Add 50 μL of water solution with 20% methanol to each well, and the resulting solution after mixing was ready for analysis.

Histopathology and immunohistochemistry analysis

Mice or cynomolgus monkeys were euthanized after the experiments. The animal heart, liver, lung and kidney were collected and fixed in 10% neutral buffered formalin for 48 h for preparing 5 μm thick sections. The sections were dewaxed, rehydrated, and stained with hematoxylin and eosin (H&E) dye. The pathological changes in the heart tissues were observed under a light microscope (×400 magnification, Olympus, Tokyo, Japan). Immunohistochemical staining was accomplished using the methods previously reported by Rocio Ramos-Medina et al. [1].

[1] Tramos-Medina R, Montes-Moreno S, Maestre L, Canamero M, Rodriguez-Pinilla M, Lazaro A, Roncador G: Immunohistochemical analysis of HLDA9 workshop antibodies against cell-surface molecules in reactive and neoplastic lymphoid tissues. Immunol Lett. 2011; 134(2):150-156.

Table S1 Serum biochemical assay for the cynomolgus monkey administrated with Zapadcine-1.

Groups No.	ALP U/L	TBIL mg/dL	AST U/L	ALT U/L	CRE mg/dL	TP g/dL	ALB g/dL	GLU mg/dL	TCHO mg/dL
Day3									
5 mg/kg	789	0.23	34	23.5	1.12	8.29	4.75	65	111
4 mg/kg	598	0.26	54	40.4	1.08	7.68	4.48	48	107
3 mg/kg	599	0.23	48	25.8	0.98	7.43	4.43	48	157
2 mg/kg	660	0.13	90	89.6	1	6.98	3.79	72	100
Day22									
2 mg/kg	563	0.14	62	58.3	1.1	6.79	3.66	46	112

Table S2 Hematology parameters of cynomolgus monkey administrated with Zapadcine-1.

Groups No.	WBC 10^9/L	RBC 10^{12}/L	hemoglobin g/L	RBC ratio %	platelet 10^9/L	Reticulocyte %	Reticulocyte 10^9/L
Day3							
5 mg/kg	8.07	6.1	142	45.1	177	0.56	34.2
4 mg/kg	10.59	5.44	132	41.1	352	0.5	27.2
3 mg/kg	18.27	5.21	125	37.8	359	0.92	47.9
2 mg/kg	18.5	5.12	126	41.4	338	1.89	96.8
Day22							
2 mg/kg	22.19	4.81	119	39.5	315	2.82	135.6

WBC, white blood cell; RBC, red blood cell.

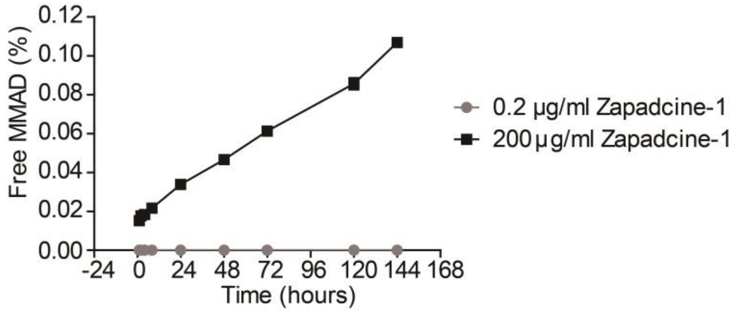

Figure S1. Identification of Zapadcine-1 stability in human plasma. Zapadcine-1 at various concentrations were incubated in human plasma for a time course and free MMAD was determined by LC/MS/MS and calculated according the following formula: Free MMAD (%) = actual generated MMAD concentration / total MMAD concentration ×100%, and total MMAD concentration = Zapadcine-1 theoretical molar concentration × DAR × MMAD molecular weight.

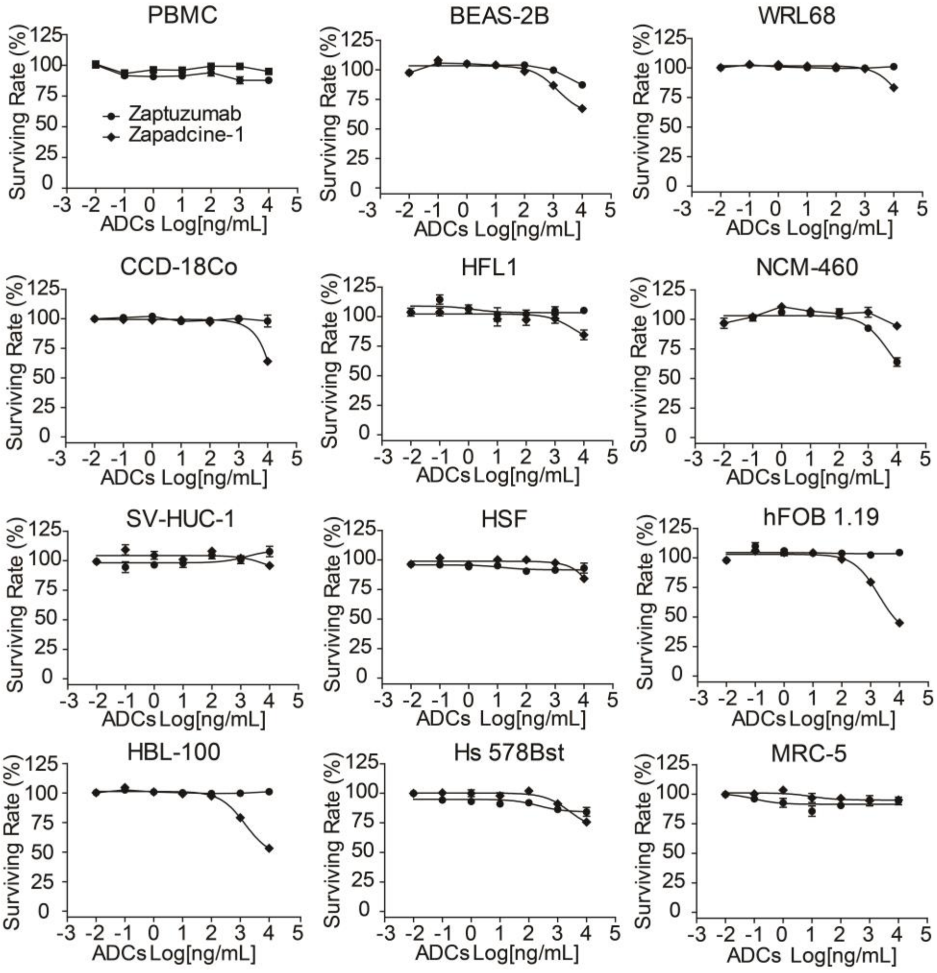

Figure S2. Cytotoxicity of Zapadcine-1 to various normal cells. Normal cells of PBMC, BEAS-2B, WRL68, CCD-18Co, HFL1, NCM-460, SV-HUC-1, HSF, hFOB 1.19, HBL-100, Hs578Bst and MRC-5 were incubated with Zapadcine-1 at indicated concentrations for a time course, respectively. The cytotoxicity was determined by CellTiter-Glo® Luminescent Cell Viability Assay according to the manufacturer's instructions. Naked Zaptuzumab was used as control. Data are presented as mean ± SD.

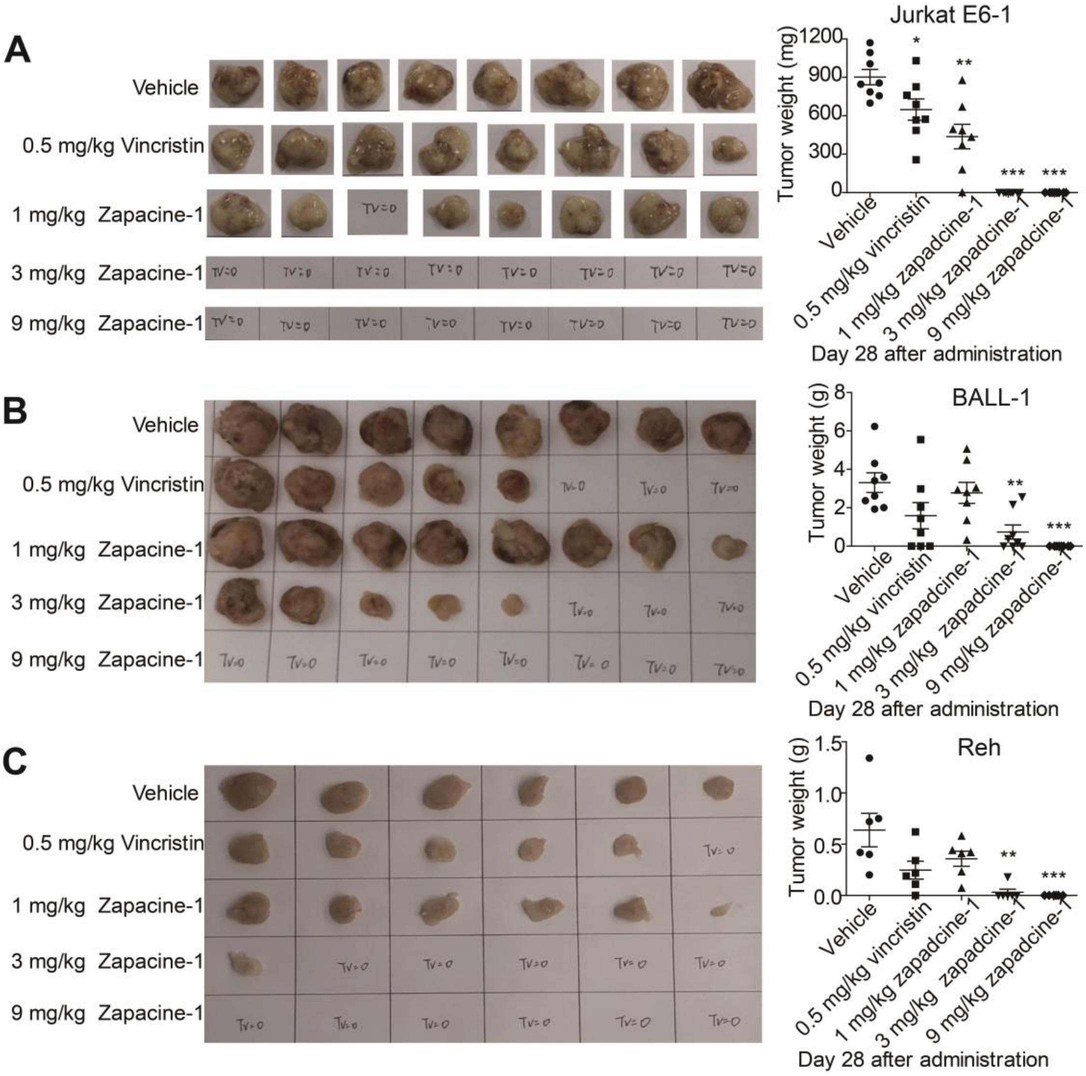

Figure S3. Efficacy of Zapadcine-1 in mouse xenografts of human acute lymphocyte leukemia cells Jurkat E6-1 (A), BALL-1 (B) and Reh (C). Representative transplanted tumor images and mouse body weights were assessed on day 28 after administration. The p values are two-tailed, $*p \leq 0.05$, $**p \leq 0.01$, $***p \leq 0.001$.

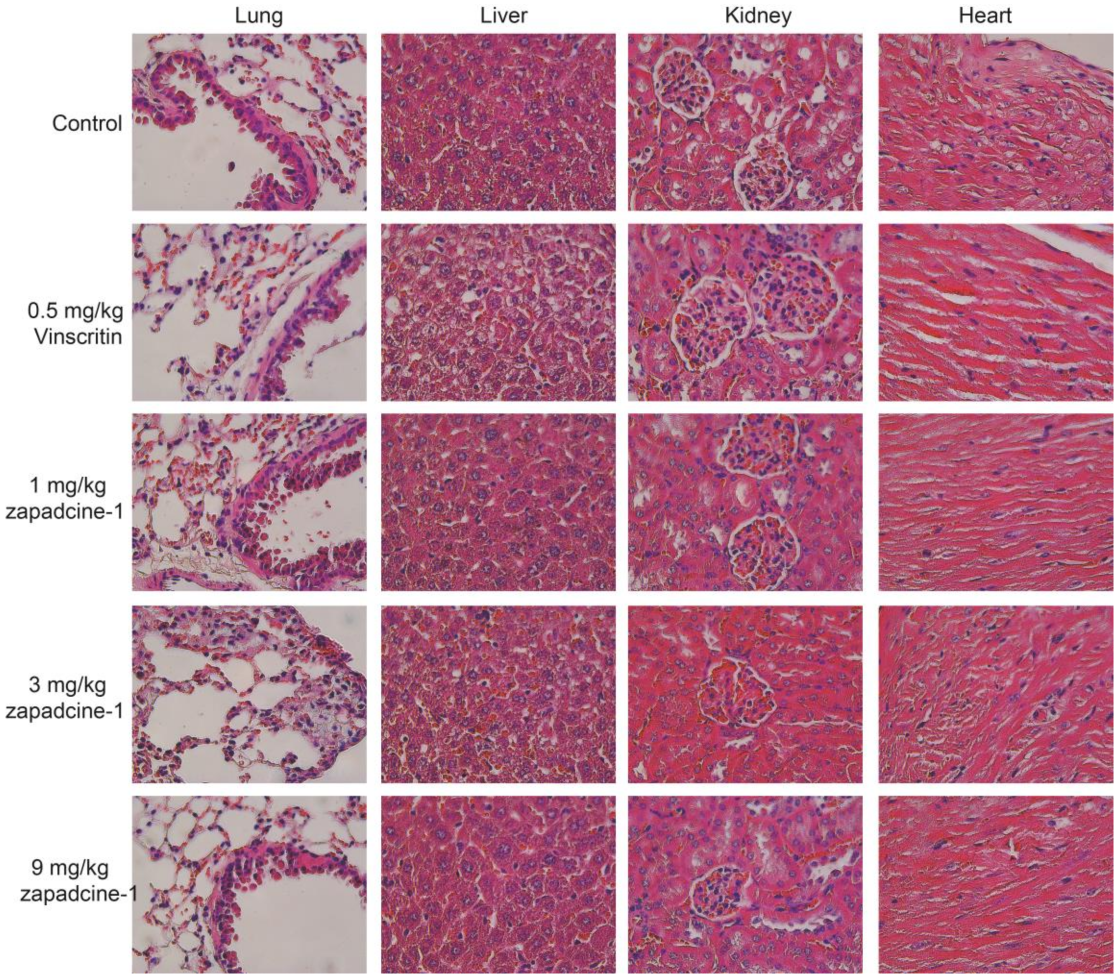

Figure S4. H&E staining assay for the evaluation of pathological changes in heart, liver, lung and kidney of human Jurkat E6-1 mouse CDX models. Magnification × 400. Scale bars = 50 μm.

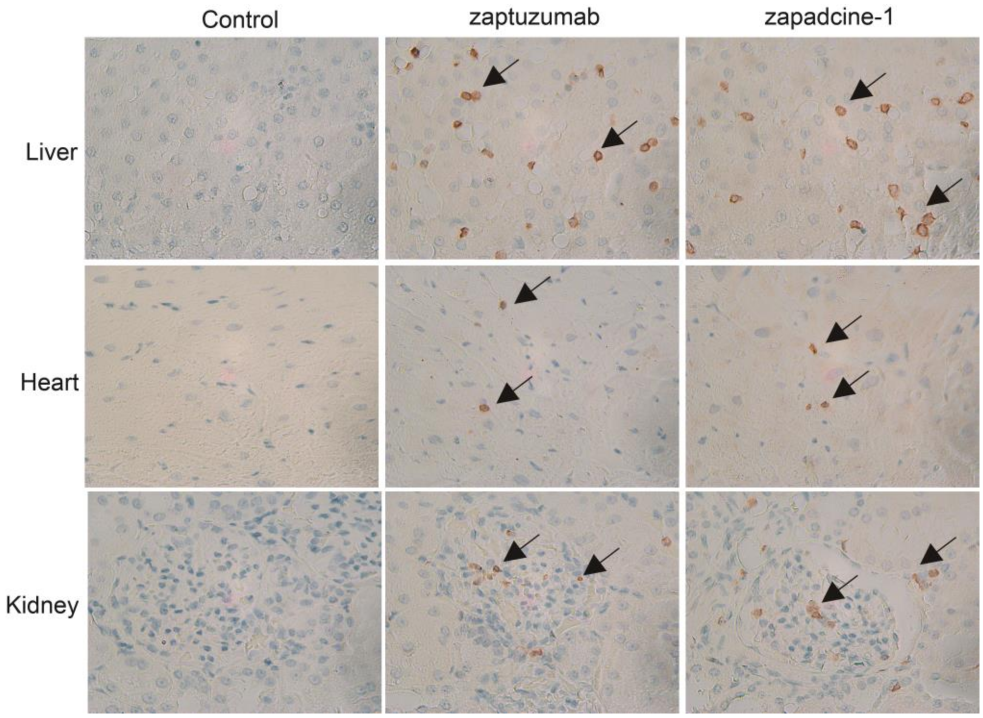

Figure S5. Immunohistochemistry assay for the DR5 expression in heart, liver and kidney of normal cynomolgus monkey. Magnification x 400. Scale bars = 50 μm.

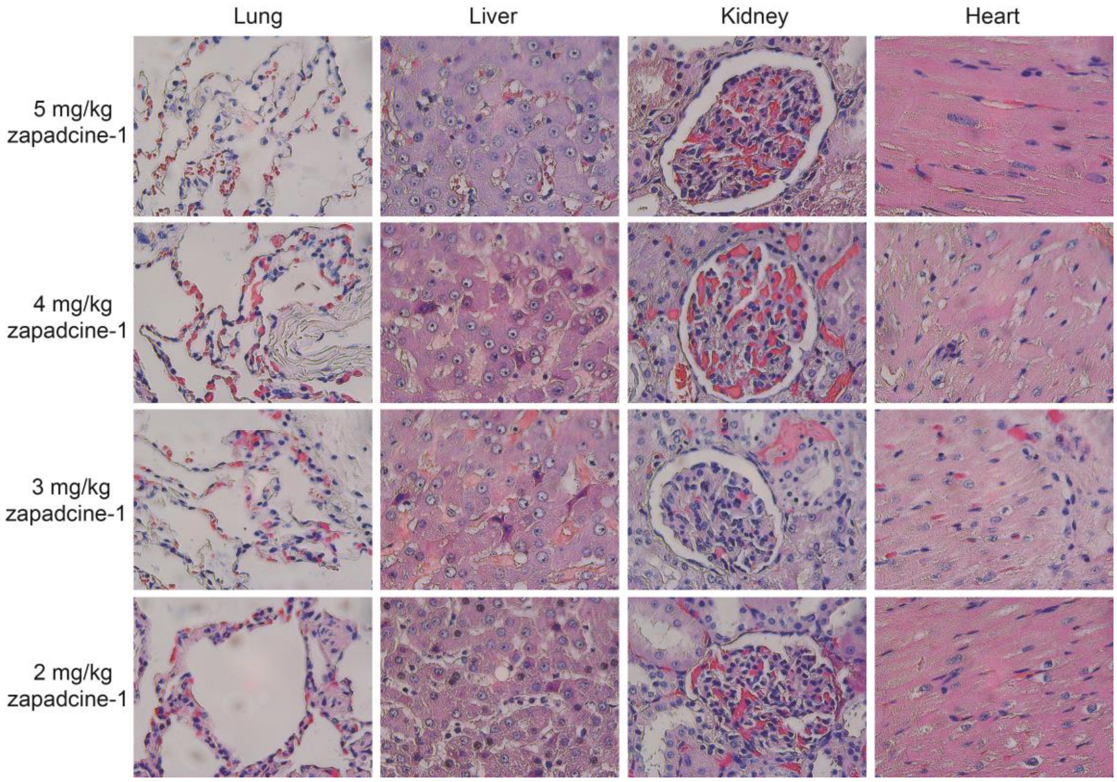

Figure S6. H&E staining assay for the evaluation of pathological changes in heart, liver, lung and kidney of normal cynomolgus monkey. Magnification × 400. Scale bars = 50 μm.
